# Extraction of Bioactive Compounds from *Prestonia mollis* Leaves and Their Impregnation into Polylactic Acid Using High-Pressure Technologies: Potential for Biomedical Application

**DOI:** 10.3390/antiox12101864

**Published:** 2023-10-15

**Authors:** Gabriel Alfonso Burgos-Briones, Lidia Verano-Naranjo, Cristina Cejudo-Bastante, Alex Alberto Dueñas-Rivadeneira, Casimiro Mantell-Serrano, Lourdes Casas-Cardoso

**Affiliations:** 1 Chemical Processes, Food and Biotechnology Department, Faculty of Mathematical, Physical and Chemical Sciences, Technical University of Manabí, Urbina Avenue and Che Guevara, Portoviejo 130105, Manabí, Ecuador; gabriel.burgos@utm.edu.ec; 2 Chemical Engineering and Food Technology Department, Faculty of Science, Wine and Agrifood Research Institute (IVAGRO), University of Cadiz, 11510 Puerto Real, Spain; lidia.verano@uca.es (L.V.-N.); cristina.cejudo@gm.uca.es (C.C.-B.); casimiro.mantell@uca.es (C.M.-S.); 3 Agroindustrial Processes Department, Faculty of Zootechnical Sciences, Technical University of Manabí, Urbina Avenue and Che Guevara, Portoviejo 130105, Manabí, Ecuador; alex.duenas@utm.edu.ec

**Keywords:** *Prestonia mollis*, pressurized liquid extraction, enhanced solvent extraction, antioxidant activity

## Abstract

Enhanced solvent extraction (ESE) and pressurized liquid extraction (PLE) have been used for the first time to obtain antioxidant compounds from *Prestonia mollis* leaves. The effects of pressure (100–250 bar), temperature (55–75 °C) and the composition of the extraction solvent (ethanol, water and hydroalcoholic mixtures) were evaluated according to multilevel factorial designs. PLE provided the largest extraction yields compared to ESE, as well as a greater impact of the operating conditions studied. The highest total phenolic content was obtained when using a hydroalcoholic mixture (CO_2_/ethanol/water 50/25/25) through ESE at 100 bar and 75 °C. The antioxidant capacity of this extract is related to higher concentration levels of the identified flavonoids: Quercetin 3-O-xylosyl-rutinoside, Kaempferol 3-(2G-apiosylrobinobioside) and Kaempferol 4′-glucoside 7-rhamnoside. This extract was tested for the supercritical impregnation of polylactic acid (PLA), which is a polymer widely used in the biomedical industry. The influence of pressure (100–400 bar), temperature (35–55 °C), amount of extract (3–6 mL) and impregnation time (1–2 h) have been evaluated. The best results were obtained by impregnating 3 mL of extract at 100 bar and 55 °C for 2 h, achieving 10% inhibition with DPPH methods. The extract presented a potentially suitable impregnation of PLA for biomedical applications.

## 1. Introduction

Natural products have been recognized since the beginning of humanity as an excellent source of bioactive compounds to treat certain diseases or to palliate specific symptoms that may affect human health [1,2]. Many of the drugs that are currently available in the market have been obtained either directly from natural products or synthesized from them. This has encouraged a recently growing interest in their properties and promoted research studies on more sophisticated and efficient extraction methods.

Ecuador is one of the countries with the greatest biodiversity in the world, with around 10% of the world’s plant species, and every year new plants are discovered and added to the long list of the species already known [3]. This makes of Ecuador an invaluable source of potentially interesting new natural products for the pharmaceutical industry.

*Prestonia mollis* (Malacapa) is an Ecuadorian plant species traditionally used for therapeutic purposes. It belongs to the *Apocinacea* family, and its branches are densely tomentose when young and glabrescent at maturity [4]. *Prestonia mollis* is considered among the species that dominate the wetland area in the Esmeraldas province on the Ecuadorian coast, and it is also very abundant in the province of Manabí. It is considered a weed in cultivated fields, and it demands laborious removal processes in certain crops such as cassava or corn, which are important sources of income for the population of Manabí. Therefore, this weed could be given an added value after its removal by using it for new purposes. According to a survey where 1593 adults were inquired, *Prestonia mollis* was popularly used in therapeutic applications for the treatment of cancer as well as for the disinfection and healing of wounds [5]. Other authors corroborate the traditional application of *Prestonia mollis* extracts to treat skin affections [3,6]. Previous research using conventional extraction methods has identified alkaloids, saponins and flavonoids as the main phytochemicals [6,7]. However, although a number of projects are being conducted in Ecuador to scientifically study its flora, as far as we know, the *Apocynaceae* family, and especially *Prestonia mollis*, has not been so far included in these studies. The scientific information available on this plant is scarce, so it is necessary to investigate the biological activity and chemical composition of the extracts.

Extraction is an important step with regard to the isolation of bioactive compounds from vegetable matter. Both the technique and the solvent are key factors to maximize a selective recovery and to avoid the extraction of undesirable substances. Nowadays, great attention is paid to green extraction technologies that allow the use of hazardous substances to be reduced or totally avoided while limiting the toll associated to solvent waste and its disposal [8]. Among these, supercritical fluid extraction (SFE), enhance solvent extraction (ESE), pressurized liquid extraction (PLE), ultrasound-assisted extraction (UAE), microwave-assisted extraction (MAE) and enzyme-assisted extraction (EAE) are all considered as prominent sustainable techniques.

Most industrial extraction processes are based on traditional systems that use organic solvents for the extraction of bioactive compounds. The environmental pollution generated by organic solvents has become a central issue in societal and political decisions. This has led to a growing demand for efficient eco-friendly extraction technologies. In the last two decades, significant advances have been made in the methods used, such as the development of ESE and PLE. The common denominator of newly emerging methods is high efficiency, low solvent consumption and significantly shorter extraction time, resulting in decreased costs, workload and impact to the environment [9].

Pressurized liquid extraction (PLE)—also known as accelerated solvent extraction (ASE), pressurized solvent extraction (PSE) or pressurized fluid extraction (PFE)— uses liquid solvents under moderately high pressure and temperature conditions that never reach their critical point levels. Under these conditions, the high diffusion coefficients and high solvent strength increase the solubility of the solutes as well as lowering the solvent viscosity and surface tension, all of which favor the extraction process [10]. Thanks to these favorable conditions, this technique can also guarantee rapid extraction rates, as the dielectric constant of the solvent is also reduced [11]. Enhanced solvent extraction (ESE), on the other hand, combines the employment of pressurized highly fluid solvents of a low viscosity, such as CO_2_, to leverage on the typical benefits of pressurized liquid solvents and the enhanced transport properties of supercritical fluids. Thus, carbon dioxide in combination with a liquid solvent (generally water and/or an alcoholic compound) forms a gas-expanded fluid that easily extracts polar compounds, such as polyphenols [12]. This process, therefore, requires a smaller amount of solvent to achieve a greater recovery of the compounds in comparison to other traditional methods or even to PLE [13].

Extraction solvent and temperature are generally the parameters with the greatest impact on these two techniques regarding the size of the extraction yields [14]. Among the potential green solvents that could be used, water stands out as an excellent option as it poses no harm to human health or the environment, besides its ready availability and low cost [15]. Ethanol is another solvent that is widely used for ESE and PLE as it is quite affordable and considered non-toxic, even though it is a flammable substance [16]. Ethanol–water mixtures have also been demonstrated to be, in some cases, more effective than other pure solvents for the extraction of polyphenols from different plant raw materials [17].

Natural phytochemical compounds are important alternatives for the development of the pharmaceutical industry through phytomedicine [18]. Polyphenols are compounds with unique chemical and biological properties, notably as bioactive antioxidants. Research has linked many classes of polyphenols to positive health outcomes and potential protection against a host of oxidative degenerative diseases, from cancer to heart disease [19,20,21]. One of the current trends with regard to the use of antioxidant compounds consists in their impregnation into polymeric matrices for biomedical applications. Supercritical impregnation has been reported as an efficient alternative process that can be successfully employed for this purpose [22,23,24]. In this procedure, supercritical CO_2_ plays several simultaneous roles as it dissolves the solutes of interest, transfers them to the polymer surface and acts as a diffusion enhancer by temporarily modifying some of the polymer properties [25]. Carbon dioxide is a gas under regular atmospheric conditions; therefore, it desorbs from the polymer and evaporates by simple depressurization, which results in a solvent-free final product.

Polylactic acid (PLA) is currently used in pharmaceutical formulations, solid dispersions for oral bio availability or drug delivery systems. PLA has good CO_2_ sorption, plasticization and swelling properties under high-pressure conditions, as reported by several authors [26,27,28], which facilitates the incorporation of drugs or active extracts. Therefore, it would be interesting to study the extraction of bioactive compounds from the Ecuadorian plant species *Prestonia mollis* and their subsequent impregnation in a polymeric matrix used in biomedicine such as PLA.

In this work, solvents generally recognized as safe (GRAS), such as CO_2_, water and/or ethanol, were used to extract and characterize bioactive compounds from *Prestonia mollis* leaves. The aim of this study is to evaluate the extraction process by non-conventional techniques, such as ESE and PLE, in terms of total extract yields and phenolic content, as well as to determine the antioxidant capacity of the extracts. The major compounds in the extracts with the highest phenolic content have also been identified by liquid chromatography. Finally, the suitability of a supercritical fluid procedure to incorporate the bioactive extracts obtained into a PLA device has been evaluated.

## 2. Materials and Methods

### 2.1. Raw Material and Chemicals

*Prestonia mollis* sp. (Malacapa) from the Pimpiguasí sector in the Abdón Calderón parish in Portoviejo canton, Manabí province, Ecuador, was the plant variety used for this study. The plants were collected in 2022 from an integral farm located by the lower section of the Chico River basin, at the coordinates −1.012624, −80.365781. The plants had been grown on a sandy loam soil under erratic rainfall conditions, and the temperature records reflected an upward trend since 2013 [29]. The leaves were dried at room temperature and crushed prior to their use.

For the extraction and impregnation processes, carbon dioxide (99.99%), purchased from Abello-Linde S.A. (Barcelona, Spain), and ethanol (>99%), supplied by Panreac (Barcelona, Spain), were used. The reagents 2,2-diphenyl-1-picrylhydrazyl (DPPH), 2,2′-Azino-bis(3-ethylbenzothiazoline-6-sulfonic acid) diammonium salt (ABTS) and Folin–Ciocalteu were purchased from Sigma-Aldrich (Steinheim, Germany). The HPLC-grade solvents (acetonitrile and formic acid) were supplied by Panreac (Barcelona, Spain).

For the impregnation process, 1.6 mm diameter PLA filaments were purchased from Mundo Reader S.L. (Madrid, Spain). The polymeric material was 100% PLA with 1.24 g/cm^3^ density, a fusion temperature of 145–160 °C and a glass transition temperature of 56–64 °C (according to the specifications provided by the manufacturer).

### 2.2. Extraction under High Pressure

**Figure 1:**
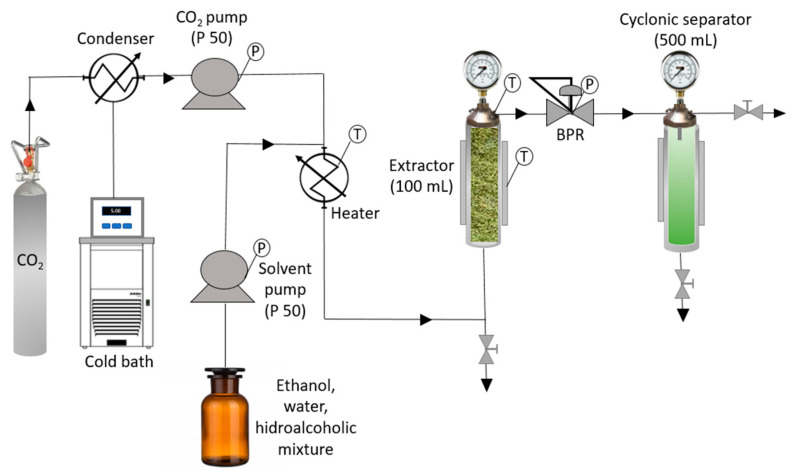
Simplified diagram of the supercritical extraction and fractionation equipment (BPR: automatic back pressure regulator, T: temperature, P: pressure).

The equipment used for the extractions was an SF100 model supplied by Thar Technologies (Pittsburgh, PA, USA), fitted with an extractor (100 mL capacity) and two pumps, both with a maximum flow rate of 50 g/min, where one was used for the CO_2_ and the other for the solvent. The pressure was controlled by means of a back-pressure regulator (BPR) and the temperature by means of a thermostatic jacket. Figure 1 shows a diagram of the system used for this research work.

The methodology used for the extractions involved the loading of the extraction vessel with approximately 7.4 g of the plant sample, which had been previously homogenized to an apparent density uniform in all the experiments. The extractions were carried out in batch mode for 3 h. Two extraction techniques were evaluated: ESE and PLE; for the ESE process, two pumps were used, one for carbon dioxide and the other for the liquid solvent, while for the PLE, only one pump for liquid solvent was employed. Pressurization was performed by pumping solvents at 10 g min^−1^ until the set pressure conditions had been achieved, when the extraction time begins. When the extraction time was completed, the extractor pressure was decreased slowly by opening BRP until reaching atmospheric conditions. The extracts were recovered in a cyclonic separator, collected in amber flasks and stored in their respective solvent medium in darkness at 4 °C before testing, in order to prevent degradation of the bioactive compounds. The experiments were carried out in duplicate for each set of conditions to validate any possible discrepancies in the measured values.

The effect on the extract when varying pressure (100–250 bar) and temperature conditions (55–75 °C) was tested. The extraction solvent was selected according to technical requirements, but economic, safety and sustainability aspects were also considered. It is well known that water, ethanol and CO_2_ are generally used in biomedical and pharmaceutical applications as they are considered GRAS solvents (generally recognized as safe) with lower toxicity than methanol or other organic solvents. Table 1 shows the composition of the solvent mixtures used in each extraction method. For the ESE, a higher proportion of CO_2_ (50% *v*/*v* of the total solvent flow) was employed. All conditions were chosen based on the results previously obtained when using other raw materials [30].

### 2.3. Characterization of the Extracts

#### 2.3.1. Total Extraction Yields

The total yields obtained were calculated as the ratio of the total dry extract mass divided by the mass of the raw material, and the results were expressed as a percentage according to Equation (1), where *m_d_* is the mass of the dry extract, and *m_l_* is the initial mass of the leaves. The extraction yields were determined in triplicate.

(1) $$\%Y=\frac{m_{d}}{m_{l}}\times 100$$

#### 2.3.2. Total Phenolic Content

The total phenolic content (TPC) of the extracts was determined through the Folin–Ciocalteu method proposed by Margrat and coworkers [31], with minor modifications. A 12.5 µL aliquot of each extract was mixed with 12.5 µL of Folin–Ciocalteu reagent and 200 µL of double-distilled water and shaken for 5 min. Then, 25 µL of sodium carbonate solution (20% *w*/*v*) was added and the mixture was shaken for another 5 min. After 60 min in the absence of light and at room temperature, the absorbance was measured at 725 nm by means of a Synergy HTX Multi-Mode Microplate Reader by BioTek Instruments (Winooski, VT, USA). The absorbance of the same mixture with ethanol instead of the extract or the standard was subtracted from the absorbance of the mixture with the extract. Gallic acid dilutions (25–300 µg/mL) were used as standards to plot a calibration line calculated according to Equation (2), where *A* is the absorbance at 725 nm, and *C* is the equivalent concentration of gallic acid expressed as µg/mL.

(2) $$A=0.0777C-0.0101\;;\;R^{2}=0.9997$$

#### 2.3.3. Antioxidant Activity

The antioxidant capacity of the extracts was determined following two free-radical-scavenging methods, 2,2-diphenyl-1-picrylhydrazyl (DPPH) [32] and 2,2′-azino-bis(3-ethylbenzothiazoline-6-sulfonic acid) (ABTS) [33].

For the DPPH assay, 293 µL of 60 µM DPPH ethanolic solution was added to 7 µL extract aliquots at different concentrations (150–9000 µg/mL) in ethanol. After 2 h of incubation at room temperature in the absence of light, the absorbance was measured at 515 nm. A control experiment was also conducted by replacing the 7 µL extract with ethanol. The percentage of inhibition of the oxidation (%*I*) was calculated by comparing the absorbance of the control (*A*_0_) against the absorbance measured after 2 h (*A_i_*) according to Equation (3).

(3) $$\%I=\frac{\left(A_{0}-A_{i\;}\right)}{A_{0}}\times 100$$

The *EC*50 is the efficient concentration that causes 50% DPPH inhibition, and this was calculated graphically using a curve by plotting the %*I* against the concentration of the extract.

On the other hand, the ABTS solution was prepared by mixing 7.4 μM ABTS diammonium salt and 2.6 μM potassium persulfate (1:1) and allowing them to react in darkness for a minimum period of 16 h. The resulting solution was diluted in ethanol up to an absorbance of 0.7 ± 0.02 at 750 nm.

For the assays, 3 µL of extract at different concentration levels (150–9000 µg/mL) in ethanol) was added to a 300 µL ABTS solution and allowed to react for 60 min in the absence of light; then, the absorbance was measured at 750 nm. The same mixture with ethanol instead of the extract aliquot was tested in the same way to be used as the control sample. The percentage of oxidation inhibition and the IC_50_ were calculated in the same way as in the DPPH assay.

#### 2.3.4. Identification of Major Phenolic Compounds

The phenolic compounds in the *Prestonia mollis* extracts were identified and quantified by ultra-high-performance liquid chromatography–electrospray ionization–time of flight–mass spectrometry (UHPLC-ESI-ToF-MS) by means of Xevo G2-S equipment supplied by Waters Corporation (Milford, MA, USA). A UPLC BEH-C18 column of 1.7 μm particle size (2.1 × 100 mm) in a thermostatic oven fixed at 45 °C was employed. A volume of 5 µL of samples was injected and a binary solvent system (A: 0.1% formic acid in water; B: 0.1% formic acid in acetonitrile) was pumped at 0.5 mL/min. The gradient of the mobile phase volumetric composition was 97% of A at the initial time, 90% of A at 3.5 min, 85% of A at 5.0 min, 80% of A at 6.5 min, 75% of A at 8.0 min, 60% of A at 9.5 min, 50% of A at 10.5 min, 25% of A at 11.5 min, 0% of A from 12.5 to 13.5 min and 97% of A at 14.0 min. The electrospray operated in negative ionization mode, with a full-scan analysis (100–1200 Da), 40 V cone voltage, 0.7 kV capillary voltage, 120 °C source temperature and 850 °C desolvation temperature.

The procedure for the identification of the flavonoid compounds was based on their mass spectra according to the bibliography. Their quantification was carried out based on a calibration line for quercetin, at concentrations from 1 to 100 µg/mL (Equation (4)), where *C* is the concentration of equivalent quercetin in µg/mL, and *y* is the peak area.

(4) $$y=-153.17C^{2}-28702C\;;\;R^{2}=0.9954$$

### 2.4. Impregnation at High Pressure

The same high-pressure equipment as described in Section 2.2 was used for the impregnation process. Firstly, a specific amount of extract (3 mL) was poured into the vessel. Then, five pieces of PLA filament (30 mm long each) were placed in a steel-made supporting basket inside the vessel to prevent any direct contact between the polymer and the extract. The impregnation processes were carried out in batch mode. The CO_2_ was first pumped at 10 g/min until the desired pressure level was reached. Then, the CO_2_ flow was cut off, and the pressure of the system was maintained throughout the impregnation time. The system was rapidly depressurized (100 bar/min) to facilitate the impregnation of the PLA filaments.

The filaments were subsequently cleaned by means of a wet napkin to remove any extract excess. Each experiment was replicated twice, and the impregnated samples were stored in the absence of light in order to prevent their deterioration prior to use.

The effect of the operating temperature (35–55 °C) and the pressure conditions (100–400 bar) on the impregnation of the hydroethanolic extract CO_2_/ethanol/water (50:25:25) into the PLA filaments was determined.

Once the best pressure and temperature conditions had been determined, the initial amount of extract was increased up to 6 mL and the impregnation time was increased by 2 h, in order to verify if there was an increment in the amount of antioxidant compounds impregnated.

#### Loadings of the Impregnated Filaments

The loadings of *Prestonia mollis* extract impregnated into the PLA filaments were established by spectrophotometric methods. In order to determine the amount of antioxidant compounds that reacted with DPPH, 4 mL of 6 × 10^−5^ M DPPH solution were kept in contact with a specific amount of impregnated filament (15 mg). After 60 min of incubation at room temperature and in the absence of light, the fall of the absorbance at 515 nm was measured.

The inhibition percentage was determined according to Equation (3), where %*I* is the percentage of inhibition, *A*_0_ is the initial absorbance of the DPPH at 515 nm and *A_i_* is its final absorbance.

### 2.5. Statistical Analysis

All the analytical determinations were performed in triplicate, and mean and standard deviations were calculated. Multilevel factorial designs were used to determine the effect of the variables on the extraction processes. The conditions for the factorial design were pressures of 100 and 250 bar; temperatures of 55 and 75 °C; and solvent composition expressed as ethanol concentrations of 0, 50 and 100%. On the basis of this design, a total of 12 experiments were carried out with two replicates for each condition and in a random way in order to minimize errors. The response variables were total extraction yield, total phenolic content and antioxidant activities using both DPPH and ABTS methods. The experimental data were processed by means of the software application STATGRAPHICS Plus 4.0 software (Warrenton, VA, USA) to carry out the analysis for the experimental design. A Pareto chart was used to represent the effect of the different factors, where the corresponding sign has been used to indicate a positive or negative effect attributable to each experimental variable.

Using the same software, the effect attributable to the impregnation time and to the amount of extract added was analyzed by ANOVA followed by a multiple-range test in order to obtain homogeneous subgroups.

The normal distribution of the data was determined by the normal probability plot.

## 3. Results

### 3.1. Enhanced Solvent Extraction

Different parameters such as pressure, temperature, and extraction solvent showed a certain effect on ESE processes. Thus, a study on the experimental conditions was required to develop an efficient ESE procedure for the successful recovery of the antioxidant compounds of *Prestonia mollis* leaves.

**Figure 2:**
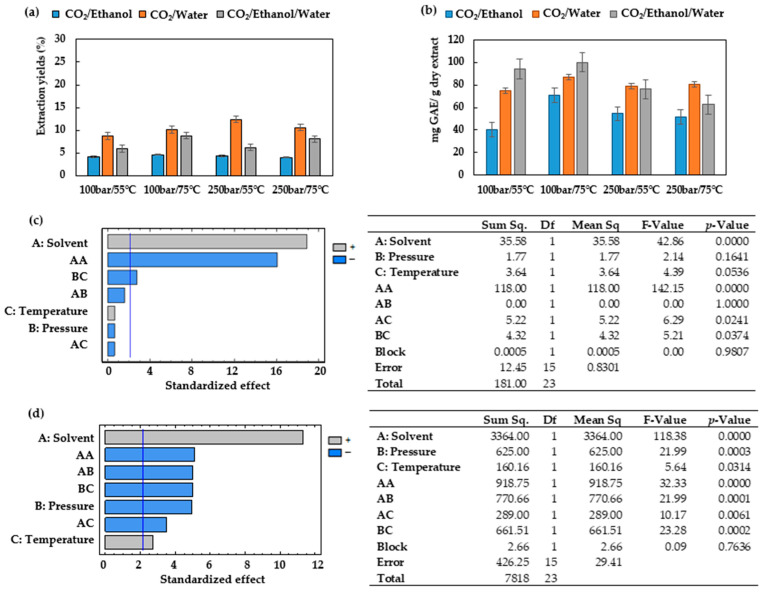
ESE experimental data when using CO_2_/ethanol (50:50), CO_2_/water (50:50) or CO_2_/ethanol/water (50:25:25) under varying operating conditions. (**a**) Total extraction yield (%) and (**b**) total phenolic content (mg GAE/g dry extract) of the *Prestonia mollis* leaf extracts (the results are expressed as their mean value ± SD). (**c**) Pareto chart and ANOVA results of the total extraction yield and (**d**) Pareto chart and ANOVA results of the total phenolic content.

The effect of the solvent composition as well as the pressure, and temperature levels was investigated according to the total extraction yields, the total phenolic content, and the antioxidant activity of the extracts. The extraction yields and the total phenolic content obtained from the ESEs are shown in Figure 2. The solvents used for the extraction of *Prestonia mollis* leaves comprised a combination of CO_2_, in every case at 50%, with water, with ethanol, or with a hydro-alcoholic mixture composed of water and ethanol.

It can be seen from Figure 2a that the mixture CO_2_/water (50:50) favored the greatest global yields (8.65–12.39% ± 1.29), whereas the use of CO_2_/ethanol (50:50) led to the lowest global yields (4.00–4.61% ± 0.46). This is explained by the fact that water contributes to disrupt the matrix–analyte interactions, which resulted in higher recoveries of the compounds from the substrate [34].

The highest phenolic content was obtained when using CO_2_/water/ethanol at 100 bar pressure (100.03 ± 4.27 mg GAE/g extract) (Figure 2b), being, therefore, the solvent CO_2_/water/ethanol (50:25:25) more selective for the recovery of the phenolic compounds in *Prestonia mollis* leaves by ESE than CO_2_/ethanol or CO_2_/water.

Since ESE can be dependent on temperature, this operating parameter must be evaluated. According to the experimental data, generally, for most of the extraction conditions, an increase in the temperature from 55 to 75 °C led to an increment of the total extraction yield regardless of the extraction solvent used (Figure 2a). The application of high temperatures favors the transfer of the analytes into the solvent and results in a more rapid and efficient extraction. Moreover, high temperatures reduce the solvent viscosity and surface tension, thus allowing the penetration of the solvent into the matrix, the wetting of the sample, and the formation of solvent cavities, all of which enhance the solubilization of the analytes in the solvent [35]. The effect of high-temperature levels is even more noticeable regarding the total phenolic content in the extracts. Thus, in general, at 100 bar, by raising the temperature within the range 55–75 °C, the total phenolic content also rises, probably because of the previously mentioned factors (Figure 2b).

Pressure helps to keep the solvent in the liquid state when temperatures above the boiling point are applied. However, pressure also favors analyte–solvent contact because it reduces the surface tension of the solvent and increases solvent penetration into matrix pores. Regarding the total extraction yields (Figure 2a) when using CO_2_/water (50:50), an increase could be observed at 55 °C as the pressure was raised from 100 to 250 bar. However, the other solvent mixtures did not lead to enhanced global extraction yields on increasing the pressure. Several studies have shown a negligible effect of pressure on ESE for the extraction of essential oils, phenolic acids and volatile compounds [34,35,36,37]. With regard to the total phenolic content obtained (Figure 2b), a negative effect of pressure could be observed when the CO_2_/water/ethanol mixture was used as the extraction solvent.

The effect of pressure, temperature, extraction solvent and their interactions was evaluated by means of a multilevel factorial design where the total extraction yields and of the total phenolic contents were considered. A Pareto chart (Figure 2c,d) was used for an evident representation, where the horizontal bars corresponding to the relevant influencing factors or interactions cross the vertical reference line. In addition, the different colors indicate whether each factor has a positive or negative influence on the response variable.

It can be seen from Figure 2c that the square of the extraction solvent and the interaction of the extraction solvent pressure have a negative effect, whereas the solvent, the temperature and the interactions of the solvent temperature have a positive effect, with a confidence level of 0.95 (*p* < 0.05). These results demonstrate how important is to keep a control on ESE operating conditions. By contrast, the Pareto chart and ANOVA shows that the total phenolic contents were significantly affected by all the variables studied (Figure 2d). The extraction solvent was the factor that most influenced the results. The correlation coefficient (R^2^) and root mean square error (RMSE) for extraction yields were 98.34% and 2.70, respectively. Similarly, the R^2^ and RMSE for the total phenolic content were 96.49% and 1.58, respectively. These results indicated a satisfactory correlation between the values.

### 3.2. Pressurized Liquid Extraction

**Figure 3:**
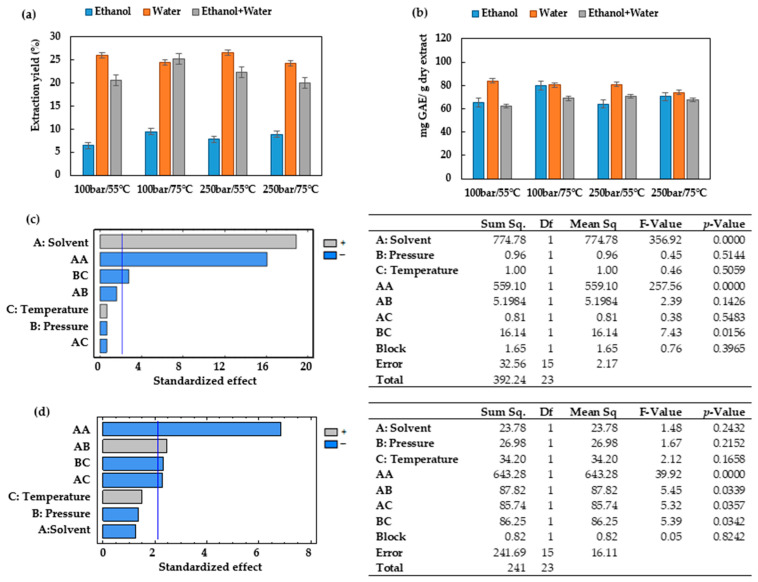
Experimental data of the PLEs when using ethanol, water and hydroalcoholic mixture ethanol/water 50:50 at varying operating conditions. (**a**) Total extraction yield (%) and (**b**) total phenolic content (mg GAE/g dry extract) of the *Prestonia mollis* leaf extracts (the results are expressed as their mean value ± SD). (**c**) Pareto chart and ANOVA results of the total extraction yield and (**d**) Pareto chart and ANOVA results of the total phenolic content.

In the present study, we have also explored the potential of PLE as a method to obtain large yields of *Prestonia mollis* leaf extracts. Three different solvents have been tested: water, ethanol and a hydroalcoholic mixture composed of ethanol and water (50:50). The same pressure and temperature conditions as for ESE have been investigated. The extraction yields and total phenolic contents resulting from the PLEs are shown in Figure 3.

A comparison between Figure 2a and Figure 3a reveals that PLE (6.41–26.66% ± 2.35) achieves higher extraction yields than ESE (3.99–12.43% ± 1.86) regardless of the solvent used. Nevertheless, a similar trend to that of ESE can be observed when water is used as the solvent, as it provides the highest extraction yields under most of the conditions studied. The hydroalcoholic mixture also achieves high extraction yields, even if they do not result in higher total polyphenolic contents (Figure 2b and Figure 3b). Only when ethanol is used can a significant increase in the extract’s phenolic content be observed in comparison to those obtained by ESE.

The effect of temperature was studied in the same range used in ESE, in order to compare the results, i.e., at 55 and 75 °C. It also must be considered that an excessively high temperature might cause the degradation of the thermolabile compounds [36,37]. Two extraction temperatures were tested, 55 °C and 75 °C. Higher temperatures were expected to enhance the extractions’ efficiency; however, it can be observed from Figure 3a that there are no large variations between extraction yields as this parameter is increased. The Pareto chart and ANOVA results (Figure 3c) show that temperature does not significantly influence on the extraction yield.

By comparing the Pareto charts and ANOVA results of the ESEs against the PLEs in terms of total extraction yields (Figure 2c and Figure 3c) and phenolic content (Figure 2d and Figure 3d), it can be seen that, in general, the PLE method is less dependent on variations of temperature and pressure. On the other hand, the choice of the extraction solvent is critical with regard to the selectivity of either method. The R^2^ and RMSE for extraction yields were 96.25% and 1.98; similarly, the R^2^ and RMSE for the total phenolic content were 97.12% and 2.08, respectively. The results indicated a satisfactory correlation between the values.

Although it is important to consider the total extraction yields and phenolic content of the extracts, we must also keep in mind that the antioxidant capacity of the extracts, as indicative of the extract’s bioactivity, is equally important. Therefore, the most relevant results concerning this biological activity are discussed in the following section.

### 3.3. Antioxidant Capacity

**Figure 4:**
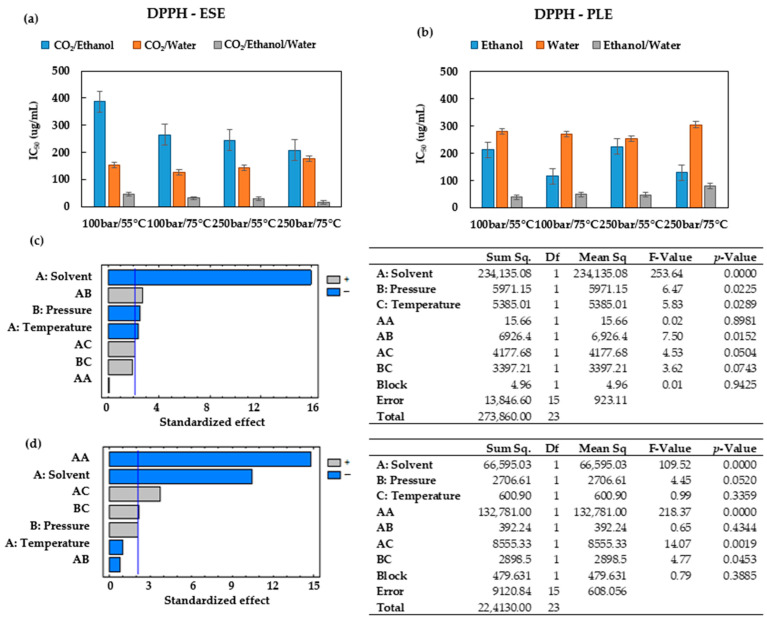
Experimental data of the DPPH conducted on ESEs and PLEs obtained under varying operating conditions. (**a**) DPPH IC_50_ of the *Prestonia mollis* leaf ESE extracts and (**b**) DPPH IC_50_ of the *Prestonia mollis* leaf PLE extracts (the results are expressed as their mean value ± SD). (**c**) Pareto chart and ANOVA results of the DPPH using ESE and (**d**) Pareto chart and ANOVA results of the DPPH using PLE.

**Figure 5:**
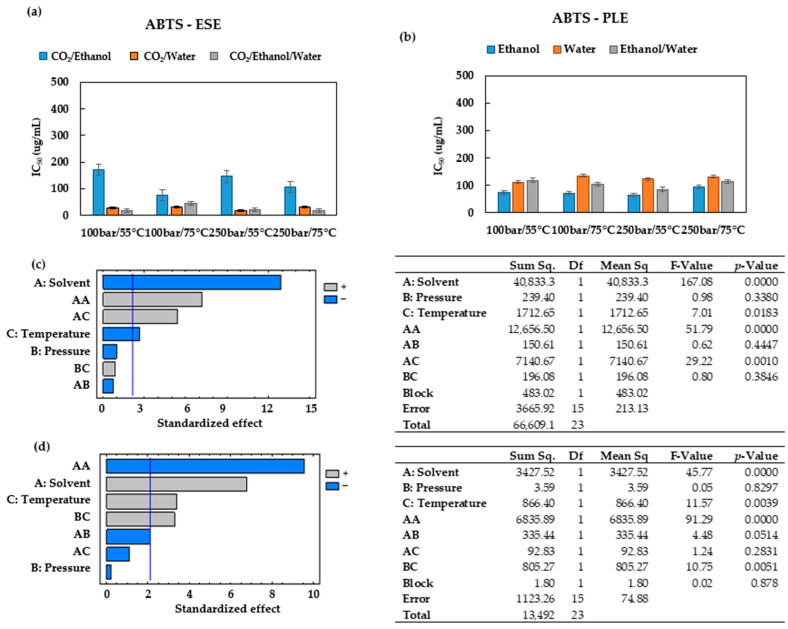
Experimental data of the ABTS conducted on ESEs and PLEs obtained under varying operating conditions. (**a**) ABTS IC_50_ of the *Prestonia mollis* leaf ESE extracts and (**b**) ABTS IC_50_ of the *Prestonia mollis* leaf PLE extracts (the results are expressed as their mean value ± SD). (**c**) Pareto chart and ANOVA results of the ABTS using ESE and (**d**) Pareto chart and ANOVA results of the ABTS using PLE.

The ability of the extracts’ bioactive compounds to scavenge free radicals was evaluated using the ABTS radical cation-based test for cation radicals and the DPPH radical-based test for stable radicals. These methods are used because they can be completed in a short time and provide a high sensitivity [38]. The results have been expressed as IC_50_ (ug/mL) vs. extraction conditions for all the solvents studied (Figure 4 and Figure 5). A lower IC_50_ value indicates a higher antioxidant activity of the extract.

A comparison of Figure 4a,b reveals that when the extractions are carried out using the hydroethanolic solvent, the highest antioxidants activities are obtained, with IC_50_ values below 80 ug/mL for all the conditions studied. When CO_2_/ethanol is used as the extraction solvent for ESEs (Figure 4a), the lowest antioxidants activities (higher IC_50_) are registered, while the extract obtained at 100 bar and 55 °C presented the highest IC_50_ among these extracts (386.9 ± 21.55 ug/mL). These results corroborate that when this solvent is used for ESEs, an increase in the operating temperature favors the extraction of the antioxidant compounds, as regardless of the pressure (100 or 250 bar), the IC_50_ values were lower when the extraction was performed at 75 °C rather than at 55 °C. The same trend was observed in the PLEs that used pure ethanol as the extraction solvent (Figure 4b). It can, therefore, be concluded that when ethanol is used as the extraction solvent, an isobaric increase in temperature favors the extraction of the antioxidant compounds from the leaves of *Prestonia mollis*.

According to the results displayed in Figure 4b, when water is the only solvent used, the variations of pressure and temperature conditions do not result in large differences with regard to the antioxidant activity of the extracts. The IC_50_ values remained rather steady at an average of 276.86 ± 18.67 ug/mL, which is noticeably higher than the IC_50_ obtained from ESEs performed using water/CO_2_ with an average value of 149.37 ± 20.18 ug/mL (Figure 4a). It appears that CO_2_ contributes to the preservation of antioxidant compounds by preventing oxidation reactions and thus obtaining better antioxidant activity values.

A comparison of Figure 4a and Figure 5a reveals that the extracts obtained when ethanol is used as the extraction solvent also have some of the highest IC_50_ values and therefore lower antioxidant activity.

Figure 5b shows that the IC_50_ values obtained through the ABTS test can reach from 63.16 up to 123.25 ug/mL across the conditions studied. This represents a narrower range of values than that obtained through the DPPH method (Figure 4b). The IC_50_ values registered by both DPPH and ABTS for the PLEs conducted using either ethanol or water (Figure 4b and Figure 5b) have been confirmed to present similar trends.

The Pareto diagrams and ANOVA results obtained for ESE and PLE using DPPH and ABTS confirm the importance of controlling the variables studied (Figure 4c,d and Figure 5c,d). The values of statistical indicators such as R^2^ and RMSE are shown in Table 2. A comparison of statistical indicators shows that there is no appreciable variation in R^2^; however, it appears that the RSME is slightly lower in the ABTS method.

### 3.4. UHPLC Identification of the Major Prestonia mollis Polyphenols

**Figure 6:**
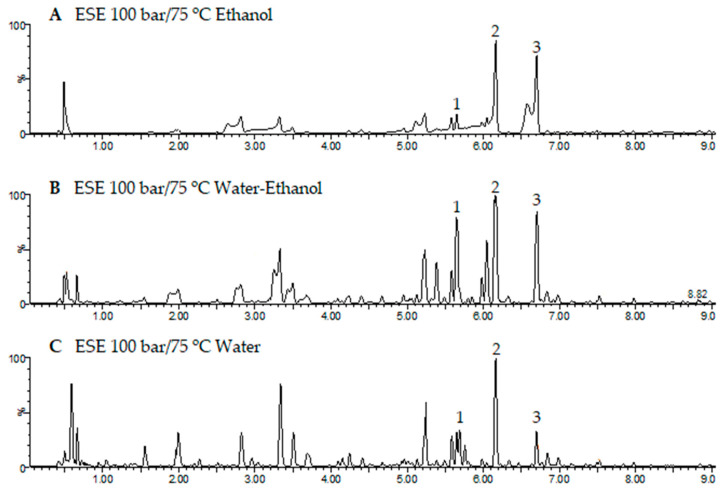
Base peak chromatograms of the extracts obtained by ESE under 100 bar and 75 °C and the three solvents considered: (**A**) ethanol + CO_2_, (**B**) water-ethanol + CO_2_ and (**C**) water + CO_2_.

The extract with the highest total phenolic content is the one extracted by ESE at 75 °C, 100 bar and CO_2_/ethanol/water (50:25:25). This has been characterized by UHPLC-ESI-ToF-MS to determine its content in major phenolic compounds. In addition, extracts obtained by ESE at the same pressure and temperature conditions but extracted with CO_2_/ethanol and CO_2_/water have been included in this analysis in order to compare the results. Figure 6 shows the chromatograms of the extracts obtained through the three different solvents. The specifications of the identified peaks and their quantifications are included in Table 3. In this table, an ANOVA analysis has been included.

### 3.5. Impregnation into the PLA Filaments

The extract that had been obtained through ESE at 100 bar, 75 °C and using the hydroalcoholic CO_2_/ethanol/water (50:25:25) mixture as solvent presented the best phenolic content results (Figure 2b). In addition, the antioxidant activity of this extract was high in comparison to the rest of the extracts obtained in this study. This extract was therefore selected to investigate the PLA impregnation process.

The effectiveness of the impregnation process depends on the nature of the polymer, the extract, the supercritical fluid, and the interactions between them. These interactions involve the solubility of the bioactive compounds in CO_2_, the capacity of the matrix to absorb CO_2_ and swell, and the extract–polymer affinity [39]. The solubility of the bioactive compounds in CO_2_, as well as the sorption and swelling of the polymer, are variables that depend on the operating conditions. Consequently, it is necessary to investigate the effect that these changing operating variables have on the impregnation yields.

**Figure 7:**
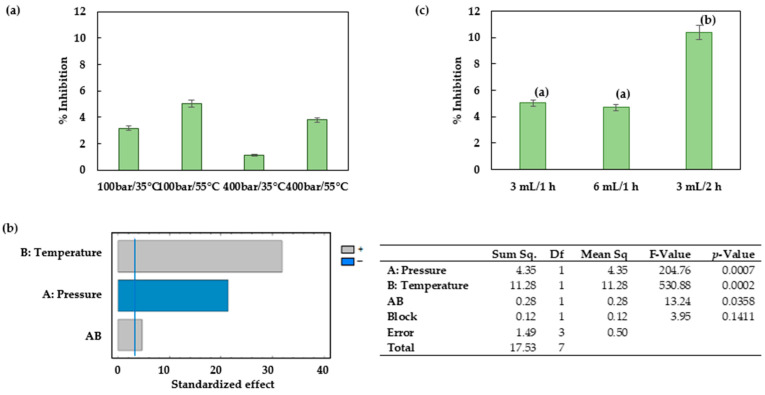
Experimental data of the PLA filament impregnations at varying operating conditions. (**a**) Inhibition percentage at different pressure and temperature levels (the results are expressed as their mean value ± SD). (**b**) Pareto chart and ANOVA results of the percentage of inhibition at different pressure and temperature levels. (**c**) Inhibition percentage when using different amounts of extract and impregnation time (the results are expressed as their mean value ± SD), where (a) and (b) indicate the homogeneous subgroups revealed by the multiple-range test.

Figure 7a shows the percentage of inhibition of the PLA filaments as a function of the pressure and temperature conditions. The best conditions were confirmed at 100 bar and 55 °C with values reaching a %I of 5.03 ± 0.37. It can be seen that when the temperature was increased, the amount of antioxidant compounds impregnated also increased, and therefore, a higher inhibition percentage was recorded. An increment in the pressure while the temperature remained steady increased the density of the CO_2_ in its supercritical state, and the analytes could present a higher affinity with the supercritical phase, which resulted in a lower impregnation of the filaments. Therefore, both of the parameters studied (pressure and temperature), as well as the interaction between them, proved to be statistically significant, as can be seen from the Pareto chart (Figure 7b). The correlation coefficient (R^2^) and RMSE for impregnation process were 97.56% and 2.15, respectively; therefore, the results indicated a satisfactory correlation between the values.

The effect of increasing the amount of extract from 3 to 6 mL was studied under the best impregnation conditions (100 bar and 55 °C). Figure 7c reveals that there was no noticeable variation in the percentage of inhibition. Apparently, 3 mL is a sufficient amount of extract to achieve a successful impregnation of the PLA filament. By contrast, an extension of the impregnation time up to 2 h did produce better results. An ANOVA followed by a multiple-range test corroborated that a greater amount of extract added to the vessel did not improve the impregnation outcome, whereas a longer impregnation time of 2 h did so (Figure 7c).

## 4. Discussion

ESE and PLE are solid extraction techniques that allow the selectivity of the extractions to be improved by means of different solvents. In ESE, water and carbon dioxide are widely studied solvents [40]. It has been reported in the related literature that water in contact with CO_2_ becomes acidic because of the formation and dissociation of carbonic acid. This acidification of liquid water contributes to breaking chemical bonds, specifically glycosidic bonds, which are characteristic of phenolic compounds, such as flavonoids, and this, in turn, increases the diffusion coefficient and promotes the release of the phenolic compounds [41]. Carbon dioxide can be easily removed after extraction by reducing the pressure to ambient temperature and is especially useful in pharmaceutical processing. The presence of water may increase the density of the fluid mixture and cause the swelling of the solutes, which consequently would improve their internal diffusion and the solubility of the compounds of interest. In addition, aqueous-organic mixtures are often used in ESE and PLE procedures to reduce the demand for organic solvents. Ethanol is also a promising solvent that can be used for the extraction of bioactive compounds. As a green solvent, it can contribute to mitigate greenhouse gas emissions, thereby reducing the environmental impact of the process [42,43,44]. Generally, the optimization of these techniques starts by choosing the most adequate extraction solvent, i.e., the one that provides the optimum analyte yield and the targeted biological activity.

On the other hand, as it has already been mentioned, an elevated pressure allows the extraction medium to be maintained in a liquid state, even when the boiling point at atmospheric pressure of the corresponding solvent has been exceeded. This elevated pressure also contributes to increasing the contact area between the matrix and the solvent by inserting the solvent into certain regions of the solid matrix that are not normally accessible to the solvent under normal pressure conditions. On its part, elevated extraction temperatures facilitate the desorption of the analytes in solid matrices and its diffusion into the solvent, and furthermore, it achieves higher values of the partition coefficients [40,45]. As a result, greater extraction yields are obtained by ESE or PLE in comparison to other conventional extraction methods.

One of the advantages of ESE in comparison to PLE is its low demand for liquid solvents. This allows the time and cost requirements to be moderated for the extract concentration stage, which in many extraction procedures may be rather long and costly. The total extraction yields from *Prestonia mollis* leaves through ESE were lower than those obtained by PLE (Figure 2a and Figure 3a). This behavior differs from reports concerning other raw materials. Thus, when the extractions were performed on mango leaves at 120 and 200 bar in the range of temperatures between 60 and 100 °C, greater total extraction yields were obtained through ESE [40]. The same behavior has been reported when using either technique for extractions from elderberry pomace at 40 °C and 209 bar [12]. Therefore, the composition of the raw material seems to be a critical aspect with regard to the total extraction yields to be expected. On the contrary, even though the total extraction yields obtained from *Prestonia mollis* leaves by ESE is discrete, this technique proves to be more selective with regard to the extraction of total polyphenols than PLE (Figure 2b and Figure 3b) in the majority of the operating conditions studied. Similarly, the total phenolic content from elderberry pomace [12] has been reported to present similar results, i.e., higher phenolic content was obtained when using ESE at 40 °C and 209 bar using CO_2_/ethanol/water.

Temperature has also been confirmed to have an effect on the total phenolic compounds. Thus, in the range 55–75 °C, higher temperature levels improved the recovery of phenolic compounds as a result of an enhanced mass transfer. Nevertheless, under certain experimental conditions, the use of 75 °C as the extraction temperature resulted in lower or unchanged phenolic yields, probably because of the presence of certain heat-sensitive polyphenols. When the ESE technique was used for the recovery of total phenolic content, temperature had a significant influence, as can be seen from Figure 2d. Under the conditions studied, the mixtures used for ESE are in a vapor–liquid equilibrium which is more strongly affected by temperature than those solvents used for PLE, all of which are in a liquid state [46].

The recovery of polyphenols from *Prestonia mollis* leaves by PLE using ethanol, water or an ethanol/water mixture (50:50 *v*/*v*) in the temperature range 55–75 °C reveals that this parameter does not have a significant effect on the process. The same occurs with the pressure factor, which in the range 100–250 bar does not have any significant influence either, whereas the pressure–solvent and pressure–temperature interaction do (Figure 3d). Both results are similar to those achieved during the extraction of the total phenolic compounds from mango leaves [40].

A previous study evaluated the recovery of total phenolic compounds from *Prestonia mollis* leaves by conventional extraction. In this case, the extraction technique used was a dynamic maceration for 4 h at room temperature using methanol as solvent. The total phenolic content obtained was 102.4 ± 2.9 mg GAE/g dry extract [6]. This result is comparable to those obtained by ESE at 100 bar and at the two temperature conditions studied but higher than the values achieved by PLE. Regarding the antioxidant activity of the extracts obtained by maceration with methanol, an IC_50_ = 500 µg extract/mL for ABTS methods and values greater than 1000 µg extract/mL according to the DPPH tests were reported [6]. The IC_50_ values reported in this work, for both extraction techniques ESE and PLE, are lower, and therefore, the extracts have greater antioxidant activity. Nevertheless, it was not possible to confirm that the antioxidant capacity of the extracts was the result of the phenolic compounds only, as the bioactivity can be affected by synergistic effects of phenolic compounds with other non-phenolic compounds, which have been shown to present antioxidant capacity.

Compounds other than phenolics must be extracted using water or the hydro-alcoholic mixture, which probably favors the antioxidant capacity of *Prestonia mollis* leaf extracts. Previous studies have suggested that the Maillard reaction inevitably occurs during extraction with hot pressurized water at high temperatures. Some Maillard reaction products, such as 5-hydroxymethylfurfural, have been shown to have a certain antioxidant capacity, and this could therefore contribute to the antioxidant activity of plant extracts [47].

Ethanolic extracts of the Ecuadorian species *Bidens pilosa* L. and *Croton floccosus* have also been evaluated in terms of antioxidant activity using the DPPH method. The IC_50_ values obtained for *Bidens pilosa* L. (239.33 µg/mL) and *Croton floccosus* (644.125 µg/mL) show that the extracts of *Prestonia mollis* obtained in the present paper exhibit a higher antioxidant activity than those obtained from these species. Malva species are widely used worldwide as traditional remedies. *Malva sylvestris* and *Malva pseudolavatera* species are the main ones sold in Ecuadorian markets. Antioxidant activity measurements by DPPH and ABTS have shown that the aqueous extract of *M. sylvestris* (IC_50_DPPH = 78.14 µg/mL; IC_50_ABTS = 166.79 µg/mL) was the most antioxidant extract, probably due to the presence of polyols [48]. These results corroborate how important it is to select the appropriate extraction technique as well as the solvent, as they have a direct influence on the bioactive compounds to be obtained.

When operating at 75 °C and 100 bar in ESEs, large contents of total phenolic compounds were obtained (Figure 2b), and the extracts exhibited low IC_50_ values (high bioactivity). Therefore, the extracts obtained at these conditions were selected for the UHPLC analysis. Figure 6 shows the base peak chromatograms of the three solvents studied. Three flavonoids were identified: Quercetin 3-O-xylosyl-rutinoside, Kaempferol 3-(2G-apiosylrobinobioside) and Kaempferol 4′-glucoside 7-rhamnoside, which were found at different concentrations depending on the solvent used for the extraction. Thus, the extracts produced using the CO_2_/ethanol/water solvent presented the highest concentration of these three flavonoids and were also the ones with the lowest IC_50_ values according to both DPPH and ABTS (Figure 4a and Figure 5a). On the other hand, the extract produced by using the CO_2_/ethanol mixture presented a higher IC_50_ value in comparison to those obtained by the extracts obtained through the other solvents and under similar pressure and temperature conditions. The CO_2_/ethanol extract presented the lowest concentration of Quercetin 3-O-xylosyl-rutinoside (538 ± 15 µg/mL).

Extraction studies on *Moringa oleifera* Lam. leaves using ESE have identified the presence of some flavanoids among which we should highlight quercetin derivates as quercetin 3-O-glucoside, quercetin malonyl glucoside, quercetin acetyl glucoside, kaempferol 3-O-glucoside and kaempferol malonyl glucoside. Not all the conditions tested extracted all the compounds, quercetin 3-O-glucoside, quercetin malonyl glucoside and kaempferol malonyl glucoside being the most representative ones and those that were recovered under every pressure and temperature configuration tested [30].

The impregnation of polymers with natural bioactive extracts using supercritical CO_2_ is a promising technique that is gaining importance with respect to biomedical applications. The extract obtained from *Prestonia mollis* leaves at 75 °C and 100 bar using CO_2_/ethanol/water presented large extraction yields of total phenolic compounds and a good antioxidant activity. Therefore, the suitability of this specific extract for the impregnation of biodegradable PLA for biomedical applications was investigated.

Figure 7 shows a successful impregnation of *Prestonia mollis* leaf extract into PLA under all the operating conditions studied. Pressure and temperature were determining factors with regard to the impregnation process with supercritical solvents as it was related to the dissolution and diffusion of the target compounds. However, the selection of pressure and temperature is a complex task, given that the most favorable conditions for the dissolution of the active compounds in the CO_2_ phase are not necessarily the best conditions to ensure a good diffusivity of the compounds and their retention within the matrix. In fact, an increase in the solubility of the target compounds (at a certain condition of pressure and temperature) may promote a high affinity of these compounds for the CO_2_ phase. Such high solubility favors an easy desorption of the target compounds during the depressurization step, which results in a lower impregnation efficiency. It is therefore necessary to find the balance between the different steps in the process with regard to solubility, diffusivity and retention of the compounds within the matrix in order to determine the partition coefficient that most favors the impregnation of the matrix. The supercritical impregnation of PLA and thermoplastic polyurethane (TPU) with active compounds have been investigated at pressure and temperature ranges from 100 to 400 bar and 35 to 55 °C, respectively. The largest olive leaf extract loadings were achieved at 250 bar and 55 °C for both polymers [49], with TPU exhibiting nearly fourfold greater OLE loadings and antioxidant activity compared to that of PLA under the optimum conditions. Another study described the impregnation of PLA filaments with mango leaf extract. In this case, the best impregnation conditions were registered at 100 bar and 55 °C [30]. These results agree with those established in this work. Under these conditions, CO_2_ presents a lower density than under the rest of the conditions studied, which results in a lower concentration of the compound in the supercritical phase and a more efficient impregnation of the polymer.

Once the most favorable pressure and temperature conditions for the process had been selected, the effect of the amount of extract was also investigated by increasing it from 3 to 6 mL. According to the results obtained, no significant differences were observed between the two amounts, and therefore, the smallest amount was considered to be adequate. However, a longer impregnation time of 2 h did actually result in significant impregnation differences. Milovanovic et al. impregnated thymol into a mixture of PLA with different amounts of poly(ε-caprolactone) (PCL) using supercritical CO_2_ [27]. They studied the effect of varying impregnation times between 1 and 15 h. According to their results, 1.5 h of impregnation time would be appropriate for the impregnation of the PLA and PCL mixture. In fact, they found out that as the impregnation time was increased up to 5 h, the impregnation loadings were greater, and then they went down as the time was increased up to 15 h. They attributed this behavior to the crystallization of the polymer matrix when saturated with supercritical CO_2_, which led to a reduction in the free space within the matrix and subsequently to a poorer impregnation.

## 5. Conclusions

ESE and PLE are two of the most widely used extraction techniques for the extraction of bioactive compounds from plant materials, as they are versatile, rapid and efficient. Given that they allow to reduce or even operate without organic solvents, they are not only more environmentally friendly but also allow to obtain more efficient extracts in terms of antioxidant activity.

This is the first research work that includes both ESE and PLE where pressure, temperature and extraction solvent are evaluated in terms of total extract yields, as well as phenolic composition and antioxidant capacity of the extracts obtained from *Prestonia mollis* leaves. A thorough analysis of the extraction results has allowed us to confirm that, even if PLE provided greater extraction yields in comparison to ESE, the total polyphenolic content of these extracts expressed as mg GAE/g dry extract did not present a similar upward trend. In relation to the antioxidant activity as measured by DPPH and ABTS, the extracts obtained by ESE at 100 bar and 75 °C exhibited higher values. The flavonoids identified have been the following: Quercetin 3-O-xylosyl-rutinoside, Kaempferol 3-(2G-apiosylrobinobioside) and Kaempferol 4′-glucoside 7-rhamnoside, all of which appeared at different concentration levels depending on the solvent used for each extraction.

The extract obtained by ESE using CO_2_/ethanol/water at 75 °C and 100 bar was applied to the supercritical CO_2_ impregnation process of PLA filaments. All the operating conditions studied achieved good impregnation results as evidenced by the percentages of inhibition achieved. The most successful impregnations were obtained at 100 bar, 55 °C for 2 h.

This study is a first step towards a better insight into the functional properties and potential applications of the extracts obtained from the Ecuadorian plant species *Prestonia mollis*. However, it would be necessary to further investigate other biological properties of these extracts, as well as other potential applications of the same.

## Figures and Tables

**Table 1:** Composition of the solvent mixtures (*v*/*v*/*v*) used for both extraction techniques.

	% CO_2_	% Ethanol	% Water
ESE	50	50	0
	50	25	25
	50	0	50
PLE	0	100	0
	0	50	50
	0	0	100

**Table 2:** Comparison of statistical analysis between DPPH and ABTS methods.

		R^2^ (%)	RMSE
DPPH	ESE	94.94	3.04
	PLE	95.93	2.46
ABTS	ESE	94.49	1.56
	PLE	95.63	0.86

**Table 3:** Composition of the solvent mixtures used for both extraction techniques.

Peak	RT	*m*/*z*	Chemical Formula	Identified Compound	Concentration (µg/mL)		
					A	B	C
1	5.65	741.186	C_32_H_38_O_20_	Quercetin 3-O-xylosyl-rutinoside	538 ± 15 ^(a)^	590 ± 15 ^(b)^	549 ± 14 ^(a)^
2	6.17	725.190	C_32_H_38_O_19_	Kaempferol 3-(2G-apiosylrobinobioside)	590 ± 18 ^(a)^	604 ± 11 ^(a)^	586 ± 18 ^(a)^
3	6.70	593.152	C_27_H_30_O_15_	Kaempferol 4′-glucoside 7-rhamnoside	585 ± 18 ^(a)^	592 ± 19 ^(a)^	539 ± 15 ^(b)^

RT: retention time; *m*/*z*: mass of the principal fragment (in negative ionization); extracts obtained by ESE under 100 bar and 75 °C and the three solvents considered: (A) ethanol + CO_2_, (B) water–ethanol + CO_2_ and (C) water + CO_2_. The results are expressed as their mean value ± SD, where (a) and (b) indicate the homogeneous subgroups revealed by the multiple-range test.

## Data Availability

All the data provided in this study are available in this article.
